# Supplementary material for: Acacia catechu Willd. Extract Protects Neuronal Cells from Oxidative Stress-Induced Damage
Source: Antioxidants (Basel). 2021 Dec 29;11(1):81. doi: 10.3390/antiox11010081 (PMC8773357; doi:10.3390/antiox11010081)
Supplement: Supplementary file 1 [file antioxidants-11-00081-s001.zip › antioxidants-1457725-supplementary.pdf]

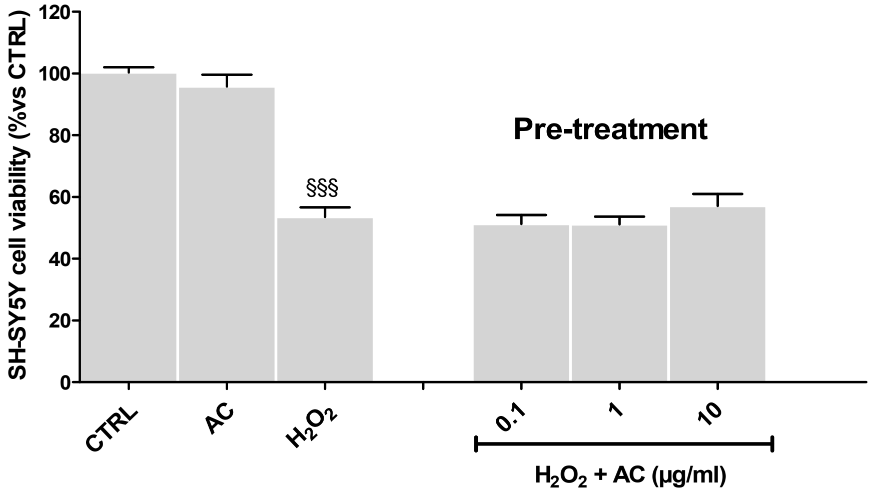

Figure S1. Effects of AC on oxidative stress (OS)-induced cytotoxicity in SH-SY5Y cells. Cells were treated with AC (0.1-10 µg/mL) according to the pre-treatment protocol described in Materials and Methods. OS was reproduced by using H_2O_2 (25 µM for 1 h + 24 h with medium). Data are reported as means ± SEMs. §§§ $p <$ 0.01, vs. untreated cells (CTRL)(ANOVA and Bonferroni post-test).

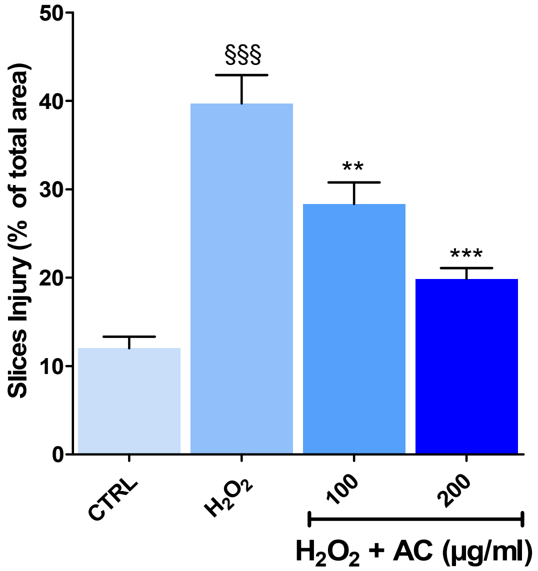

Figure S2. Effects of AC on OS-induced reduction in rat brain slices viability. Slices were incubated with artificial cerebrospinal fluid (ACSF) (controls) or ACSF + AC for 1 h. Afterward, AC was maintained and H_2O_2 (5 mM for 1 h) was added. Viability was expressed as area of the injury vs total area of the slice, as calculated by using ImageJ software. Data are reported as means ± SEMs. §§§ $p < 0.001$, vs. untreated slices (CTRL); ** p < 0.01, *** $p < 0.001$ vs. H_2O_2 (ANOVA followed by Bonferroni post-test).

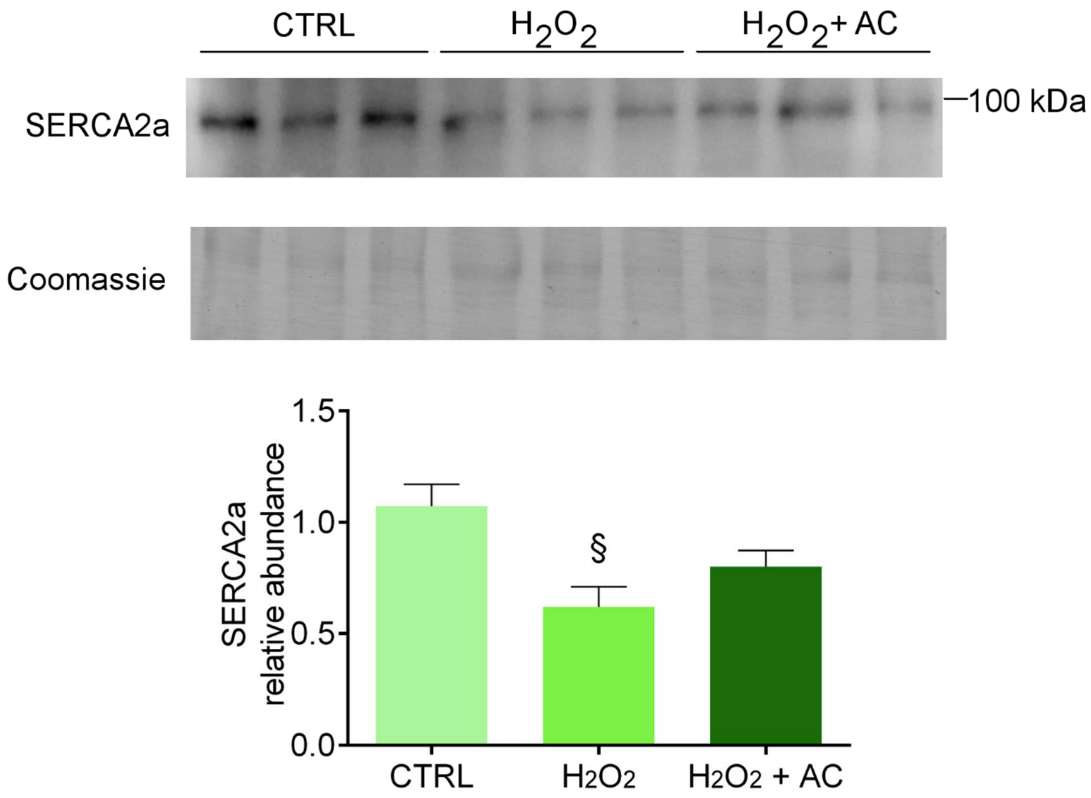

Figure S3. The H_2O_2-induced depletion of SERCA2a is only partially rescued by AC treatment. Protein lysates derived from rat brain slices (treated as described in Materials and Methods) were analyzed by WB using antibodies against SERCA2a. The upper panel shows a representative WB and the Coomassie blue staining of the membrane, while the bar diagram shows the densitometric analysis of SERCA2a immunoreactive bands normalized to the optical density of the corresponding Coomassie-stained lane. Data are reported as means ± SEMs. § p <0.05 vs CTRL (ANOVA followed by Holm Sidak's multiple comparison test).

Table S1 - Altered proteins in rat brain slices treated with H_2O_2 or H_2O_2+AC 200 μg/mL.

			mean value			SEMs			p-value	
**Gene name**	**Var ID (Primary)**	**Protein name**	**CTRL**	**H_2O_2**	**H_2O_2+AC**	**CTRL**	**H_2O_2**	**H_2O_2+AC**	**CTRL vs H_2O_2**	**H_2O_2 vs H_2O_2+AC**
Usp5	D3ZVQ0	Ubiquitin carboxyl-terminal hydrolase	1,00	1,36	1,11	0,09	0,00	0,09	0,0003	0,0340
H2afx	D3ZXP3	Histone H2A	1,00	1,19	1,06	0,04	0,04	0,05	0,0140	0,0407
Syngr3	D4ABK1	Synaptogyrin 3	1,00	1,33	1,11	0,03	0,09	0,11	0,0103	0,0477
Sh3gl1	O35964	Endophilin-A2	1,00	1,30	0,99	0,05	0,04	0,01	0,0030	0,0067
Syn1	P09951	Synapsin-1	1,00	1,15	0,94	0,05	0,02	0,07	0,0386	0,0278
Cox4i1	P10888	Cytochrome c oxidase subunit 4 isoform 1, mitochondrial	1,00	1,17	1,01	0,04	0,01	0,03	0,0029	0,0034
Fh	P14408	Fumarate hydratase, mitochondrial	1,00	1,46	1,12	0,07	0,07	0,10	0,0022	0,0150
Anxa5	P14668	Annexin A5	1,00	1,24	0,88	0,04	0,02	0,16	0,0457	0,0229
Sptan1	P16086	Spectrin alpha chain, non-erythrocytic 1	1,00	1,23	1,05	0,04	0,04	0,07	0,0202	0,0265
Gria2	P19491	Glutamate receptor 2	1,00	1,41	1,13	0,03	0,08	0,13	0,0026	0,0182
Atp5pf	P21571	ATP synthase-coupling factor 6, mitochondrial	1,00	1,38	1,12	0,05	0,01	0,10	0,0111	0,0330
Pcmt1	P22062	Protein-L-isoaspartate(D-aspartate) O-methyltransferase	1,00	1,27	1,06	0,07	0,04	0,09	0,0238	0,0401
Fabp7	P55051	Fatty acid-binding protein, brain	1,00	1,24	1,12	0,02	0,05	0,04	0,0016	0,0394
Ppp1cb	P62142	Serine/threonine-protein phosphatase PP1-beta catalytic subunit	1,00	1,20	1,05	0,07	0,04	0,07	0,0254	0,0340
Add2	Q05764	Beta-adducin	1,00	1,17	1,00	0,03	0,05	0,05	0,0269	0,0269
Cnrip1	Q5M7A7	CB1 cannabinoid receptor-interacting protein 1	1,00	1,22	1,01	0,06	0,05	0,08	0,0483	0,0483
Ptprz1	Q62656	Receptor-type tyrosine-protein phosphatase zeta	1,00	1,12	0,97	0,03	0,04	0,04	0,0253	0,0160
Septin9	Q9QZR6	Septin-9	1,00	1,33	0,93	0,14	0,07	0,27	0,0476	0,0150
Ckm	P00564	Creatine kinase M-type	1,00	1,48	1,47	0,10	0,18	0,09	0,0186	0,5910
Thy1	P01830	Thy-1 membrane glycoprotein	1,00	1,13	1,06	0,03	0,02	0,05	0,0383	0,2885
Napa	P54921	Alpha-soluble NSF attachment protein	1,00	1,12	1,12	0,03	0,01	0,05	0,0169	0,4522
Idh2	P56574	Isocitrate dehydrogenase [NADP], mitochondrial	1,00	1,67	1,32	0,14	0,12	0,12	0,0080	0,0539
Pcp4	P63055	Calmodulin regulator protein PCP4	1,00	0,61	0,72	0,10	0,04	0,24	0,0156	0,4304

Data are reported as means ± SEMs. The *p*-value was calculated according to ANOVA followed by Dunnet's post hoc test. In light blue, proteins whose abundance was not reverted by AC ($p > 0.05$).

Table S2. Enrichment analysis of proteins whose expression whose reverted by AC in rat brain slices. Statistically significant Gene Ontology (GO) terms are reported in dark blue

Term	p-value	Adjusted p-value	Odds ratio	Combined score	Genes
**exocytic vesicle membrane (GO:0099501)**	**0,001**	**0,049**	**39,32**	**255,68**	**SYNGR3;SYN1**
**synaptic vesicle membrane (GO:0030672)**	**0,001**	**0,049**	**39,32**	**255,68**	**SYNGR3;SYN1**
**extracellular membrane-bounded organelle (GO:0065010)**	**0,003**	**0,050**	**28,37**	**167,01**	**PCMT1;SPTAN1**
**extracellular vesicle (GO:1903561)**	**0,003**	**0,050**	**26,88**	**155,43**	**PCMT1;SPTAN1**
mitochondrial membrane (GO:0031966)	0,004	0,051	6,99	38,76	ATP5PF;COX4I1;OGDH;SDHA
oxoglutarate dehydrogenase complex (GO:0045252)	0,007	0,058	184,89	917,89	OGDH
cytoskeleton (GO:0005856)	0,009	0,058	5,42	25,37	SEPTIN9;SPTAN1;SYN1;ADD2
PTW/PP1 phosphatase complex (GO:0072357)	0,010	0,058	123,25	570,56	PPP1CB
mitochondrial inner membrane (GO:0005743)	0,011	0,058	7,25	33,00	ATP5PF;COX4I1;SDHA
microtubule cytoskeleton (GO:0015630)	0,011	0,058	7,19	32,52	CCT7;SEPTIN9;SPTAN1
protein serine/threonine phosphatase complex (GO:0008287)	0,011	0,058	105,63	475,00	PPP1CB
neuron to neuron synapse (GO:0098984)	0,011	0,058	105,63	475,00	GRIA2
organelle inner membrane (GO:0019866)	0,012	0,058	6,87	30,26	ATP5PF;COX4I1;SDHA
mitochondrial matrix (GO:0005759)	0,012	0,058	6,83	29,97	FH;IDH2;OGDH
mitochondrial respiratory chain complex IV (GO:0005751)	0,014	0,059	82,15	351,18	COX4I1
asymmetric synapse (GO:0032279)	0,015	0,059	11,65	49,07	GRIA2;ADD2
postsynaptic density (GO:0014069)	0,016	0,059	11,22	46,47	GRIA2;ADD2
focal adhesion (GO:0005925)	0,016	0,059	6,12	25,14	PPP1CB;ANXA5;THY1
cell-substrate junction (GO:0030055)	0,017	0,059	6,01	24,40	PPP1CB;ANXA5;THY1
septin cytoskeleton (GO:0032156)	0,019	0,060	56,86	224,10	SEPTIN9
septin ring (GO:0005940)	0,019	0,060	56,86	224,10	SEPTIN9
proton-transporting ATP synthase complex (GO:0045259)	0,021	0,061	52,80	204,47	ATP5PF
membrane raft (GO:0045121)	0,022	0,061	9,47	36,25	THY1;ADD2
mitochondrial proton-transporting ATP synthase complex (GO:00057	0,025	0,067	43,47	160,53	ATP5PF
microtubule (GO:0005874)	0,027	0,067	8,46	30,66	CCT7;SEPTIN9
AMPA glutamate receptor complex (GO:0032281)	0,028	0,067	38,89	139,57	GRIA2
anchored component of external side of plasma membrane (GO:0031	0,028	0,067	38,89	139,57	THY1
intracellular organelle lumen (GO:0070013)	0,029	0,068	3,78	13,34	FH;IDH2;OGDH;SPTAN1
intrinsic component of external side of plasma membrane (GO:003123	0,033	0,074	32,12	109,50	THY1
excitatory synapse (GO:0060076)	0,034	0,075	30,78	103,70	GRIA2
vesicle (GO:0031982)	0,040	0,081	6,78	21,89	PCMT1;SPTAN1
dendrite membrane (GO:0032590)	0,040	0,081	26,38	85,02	THY1
non-motile cilium (GO:0097730)	0,044	0,084	23,82	74,49	SEPTIN9
ionotropic glutamate receptor complex (GO:0008328)	0,044	0,084	23,82	74,49	GRIA2
polymeric cytoskeletal fiber (GO:0099513)	0,050	0,092	5,97	17,94	CCT7;SEPTIN9
neuromuscular junction (GO:0031594)	0,052	0,094	19,95	59,04	SYNGR3
dendrite (GO:0030425)	0,055	0,096	5,66	16,45	GRIA2;THY1
vacuolar membrane (GO:0005774)	0,061	0,104	16,77	46,87	NAPA
anchored component of plasma membrane (GO:0046658)	0,062	0,104	16,40	45,48	THY1
tertiary granule lumen (GO:1904724)	0,074	0,121	13,66	35,52	SPTAN1
specific granule lumen (GO:0035580)	0,083	0,132	12,09	30,04	SPTAN1
glutamatergic synapse (GO:0098978)	0,092	0,143	10,84	25,83	SH3GL1
cation channel complex (GO:0034703)	0,097	0,147	10,24	23,84	GRIA2
plasma membrane raft (GO:0044853)	0,109	0,161	9,10	20,18	ADD2
platelet alpha granule (GO:0031091)	0,119	0,171	8,27	17,63	STXBP1
mitochondrial envelope (GO:0005740)	0,163	0,231	5,83	10,57	OGDH
neuron projection (GO:0043005)	0,182	0,252	2,70	4,59	GRIA2;THY1
endocytic vesicle membrane (GO:0030666)	0,199	0,267	4,67	7,54	GRIA2
specific granule (GO:0042581)	0,202	0,267	4,62	7,39	SPTAN1
tertiary granule (GO:0070820)	0,206	0,268	4,50	7,11	SPTAN1
intracellular non-membrane-bounded organelle (GO:0043232)	0,219	0,279	1,96	2,97	SDHA;SYN1;ADD2
endocytic vesicle (GO:0030139)	0,234	0,292	3,90	5,67	GRIA2
lytic vacuole (GO:0000323)	0,265	0,326	3,36	4,45	USP5
cilium (GO:0005929)	0,284	0,342	3,10	3,90	SEPTIN9
bounding membrane of organelle (GO:0098588)	0,292	0,345	1,93	2,38	NAPA;GRIA2
integral component of plasma membrane (GO:0005887)	0,333	0,387	1,53	1,68	GRIA2;PTPRZ1;THY1
actin cytoskeleton (GO:0015629)	0,360	0,403	2,31	2,36	SEPTIN9
secretory granule lumen (GO:0034774)	0,360	0,403	2,31	2,36	SPTAN1
collagen-containing extracellular matrix (GO:0062023)	0,416	0,450	1,91	1,68	ANXA5
cytoplasmic vesicle membrane (GO:0030659)	0,416	0,450	1,91	1,68	GRIA2
lysosome (GO:0005764)	0,492	0,524	1,52	1,08	USP5
nucleolus (GO:0005730)	0,649	0,676	0,97	0,42	SDHA
nuclear lumen (GO:0031981)	0,655	0,676	0,96	0,41	SDHA
intracellular membrane-bounded organelle (GO:0043231)	0,772	0,785	0,78	0,20	PPP1CB;PCP4;USP5;OGDH;SPTAN1;PFKP
nucleus (GO:0005634)	0,785	0,785	0,75	0,18	PPP1CB;PCP4;USP5;OGDH;PFKP
